# Clinical Utility of Whole-Exome Sequencing in a Consanguineous Family with UNC80-related Neurodevelopmental Disorder: A Case Series and Review of the Literature

**DOI:** 10.7759/cureus.97861

**Published:** 2025-11-26

**Authors:** Duaa M Alshabani, Lama A Aljilani, Anas S Alyazidi, Osama Y Muthaffar, Faris A Althubaiti, Ahmed K Bamaga

**Affiliations:** 1 Medicine, King Abdulaziz University Faculty of Medicine, Jeddah, SAU; 2 Pediatrics, King Abdulaziz University Faculty of Medicine, Jeddah, SAU

**Keywords:** epilepsy, pediatric, saudi arabia, unc80 gene, whole exome sequencing

## Abstract

The *UNC79 *and *UNC80* proteins form key regulatory subunits of the sodium leak channel, non-selective (NALCN), a channel fundamental to maintaining neuronal excitability. The *UNC80* gene, located on chromosome 2 at the 2q34 locus, encodes a critical component of this channel complex, contributing to its permeability to Na⁺, K⁺, and Ca²⁺ ions. Mutations in this gene are presumed to be linked to symptoms such as hypotonia, intellectual disability, seizures, and more. With fewer than 50 cases reported, the prevalence remains unknown. In this study, we present three novel cases. Two of the cases involve siblings: a seven-year-old girl and a two-year-old boy, both diagnosed through whole-exome sequencing with a homozygous mutation in the UNC80 gene. This mutation results in a clinical phenotype that includes seizure disorder, infantile hypotonia, global developmental delay, and failure to thrive. Additional manifestations in the girl included pulmonary stenosis and an atrial septal defect. The third case is their elder brother, diagnosed at the age of 17, although he initially received a diagnosis of spastic quadriplegic cerebral palsy earlier in his disease course. In this report, we highlight the diagnostic findings, previously published case reports, and management strategies for this rare genetic disorder.

## Introduction

Molecular and physiological рole of the NALCN-*UNC80* complex

Neuronal excitability and the maintenance of the resting membrane potential are regulated by the highly conserved, voltage-insensitive sodium (Na⁺) leak channel, non-selective (NALCN). These physiological properties depend on ion gradients and membrane permeability to individual ions [1]. The channel's function is primarily regulated by its associated proteins *UNC79* and *UNC80*, located on chromosome 2 at the 2q34 locus [2]. Serving as essential scaffolding and stabilizing subunits that ensure proper channel assembly, membrane localization, and G protein-coupled receptors (GPCR)-mediated gating, *UNC79* and *UNC80* are critical for normal NALCN activity. The *UNC80* gene encodes a large protein predominantly expressed in neural tissue. As a key component of the trimeric, voltage-independent NALCN complex, *UNC80* contributes to the channel's permeability to Na⁺, potassium (K⁺), and calcium (Ca²⁺) ions, thereby helping modulate baseline neuronal excitability [3].

Clinical phenotype of *UNC80*-related disorder

Pathogenic variants in *UNC80* are associated with a severe neurodevelopmental disorder characterized by early-onset feeding difficulties, hypotonia, growth retardation, intellectual disability, and congenital encephalopathy, typically without structural brain malformations [4]. Refractory seizures beginning in infancy or early childhood have also been widely documented [5,6]. Additional reported features include clubfoot, joint contractures, scoliosis, recurrent infections, sleep disturbances, and constipation [7]. Although the true prevalence remains unknown, fewer than 50 affected individuals have been described in the literature to date [7].

Diagnostic considerations, prevalence, and management

A diagnosis of *UNC80*-related disorder should be considered in individuals presenting with global developmental delay (often with severe motor impairment), absent speech, neonatal hypotonia, profound intellectual disability, strabismus, limb dyskinesia, postnatal growth failure, postnatal microcephaly in some cases, sleep disturbances, irritability, constipation, and early-onset seizures [4,5,7]. Molecular confirmation typically demonstrates biallelic pathogenic or likely pathogenic *UNC80* variants resulting in loss of *UNC80* protein function [7]. Management is supportive and symptom-driven, including feeding interventions (with gastrostomy tube placement as needed) [8], standard therapeutic strategies for seizures, irritability, spasticity, and dyskinesia, and comprehensive developmental and educational support. Sleep disturbances may respond to melatonin or risperidone, while constipation is managed with standard approaches. Orthopedic complications may require bracing or corrective surgery. Ongoing monitoring should assess growth and nutrition, feeding safety, gastrointestinal function, seizure control, vision, musculoskeletal health, mobility, behavior, and developmental progress at least annually [7].

## Case presentation

Case one

**Figure 1:**
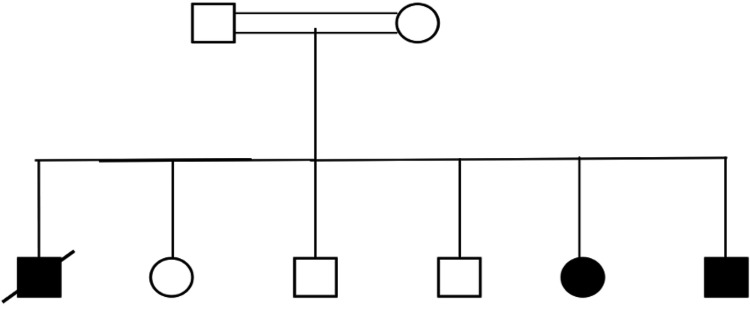
The pedigree of the affected family

This is a seven-year-old Yemeni girl who had a history of seizures, infantile hypotonia, global developmental delay, and failure to thrive. Her parents were consanguineous. She was born naturally at full term via vaginal delivery, and her mother gave no history of being exposed to medications or teratogenic pathogens while she was pregnant. Family history was significant for multiple first-degree consanguinities, developmental delays, and hypotonia (Figure 1).

**Figure 2:**
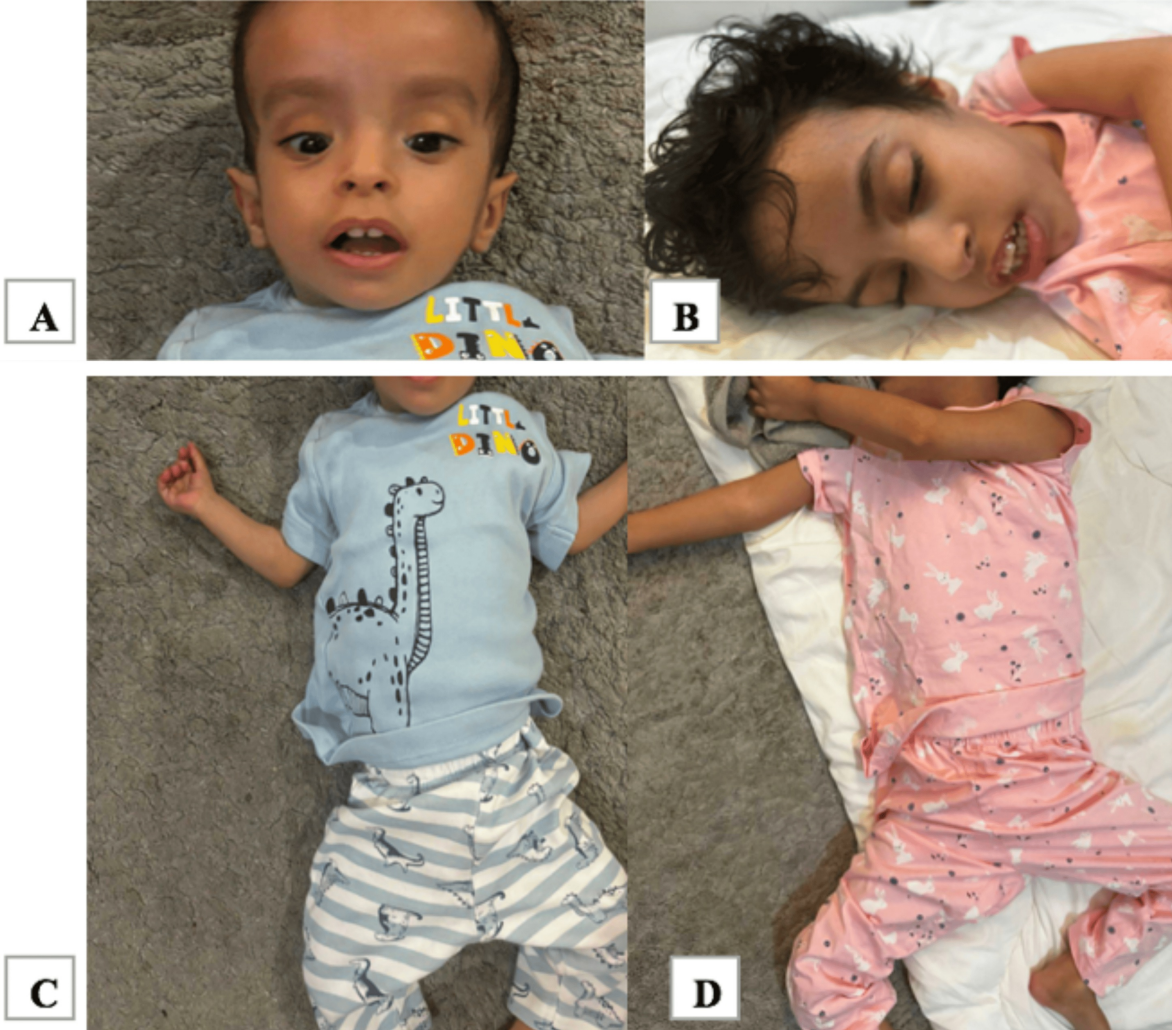
(A) Dysmorphic features include brachycephaly with strabismus, frontal bossing, triangular face with high forehead, deep-set eyes, and large ears. (A,B) Showing facial hypotonia (open-mouth appearance) and thin upper lip vermillion. (B) presenting with long face, high forehead, (C,D) Showing frog leg posture.

Upon her initial presentation, she presented with status epilepticus to our pediatric emergency department (ED). The family reported that their child experienced five to seven seizures per day over the past four days, each lasting approximately one minute and accompanied by agitation and lack of sleep. Additionally, she exhibited a decrease in oral intake. On physical examination, she appeared agitated and irritable, with dysmorphic features and severe hypotonia with hyperreflexia (Figure 2).

**Figure 3:**
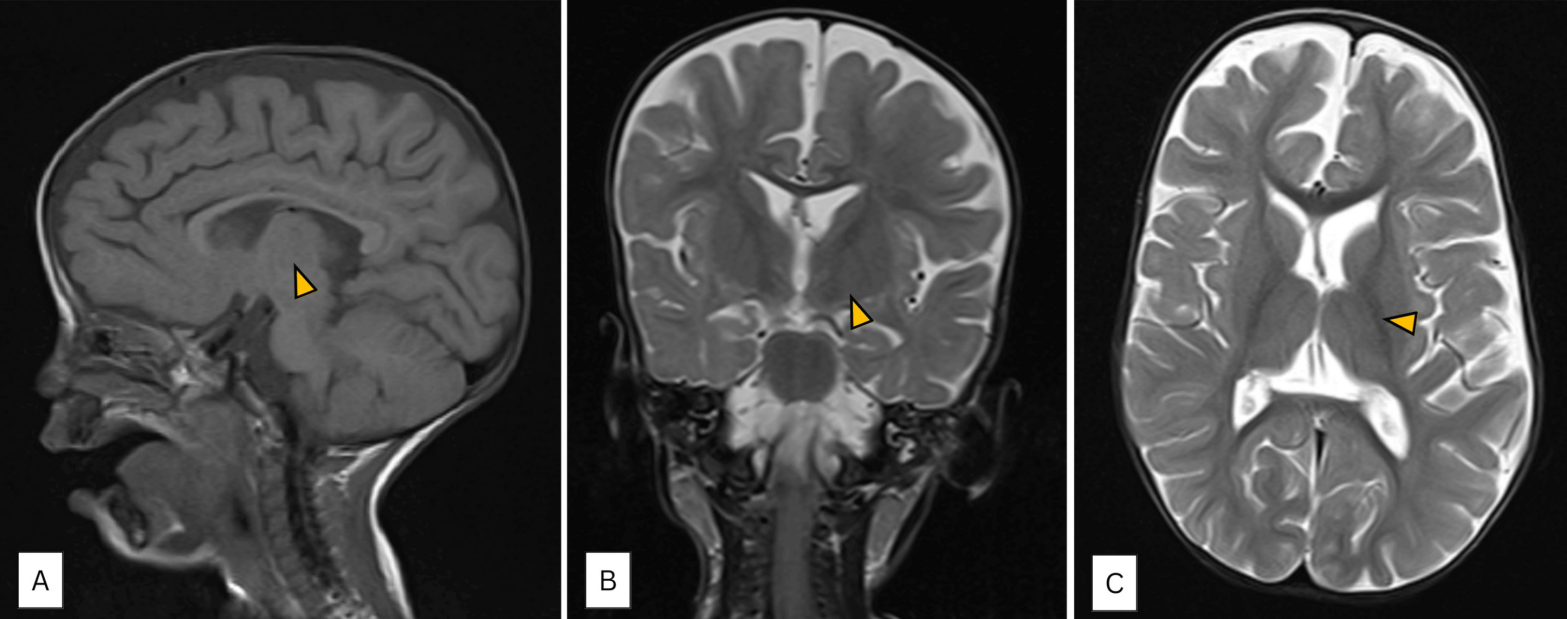
Magnetic resonance imaging (MRI) of the brain in a patient with suspected neurodevelopmental disorder. (A) Sagittal T1-weighted image demonstrates normal brain midline structures with no apparent malformation or volume loss; the corpus callosum appears intact (arrowhead). (B) Coronal T2-weighted image shows symmetric ventricular configuration and preserved cortical and subcortical architecture without evidence of focal lesions or asymmetry (arrowhead). (C) Axial T2-weighted image reveals symmetric lateral ventricles and homogeneous brain parenchymal signal intensity, with no signs of ventriculomegaly, focal abnormality, or diffusion restriction (arrowhead).

The chest examination revealed equal bilateral air entry, normal heart sounds with a pansystolic murmur, warm peripheries, and a capillary refill time of less than two seconds. Her brain magnetic resonance imaging (MRI) revealed prominent superficial brain cortical sulci (Figure 3).

Echocardiography showed pulmonary stenosis and an atrial septal defect. The patient's laboratory investigations included complete blood count, venous blood gas analysis, electrolyte panel, liver function tests, and renal function tests that were all within normal limits. Whole-exome sequencing (WES) was performed in an outside hospital, which confirmed a homozygous frameshift mutation on the *UNC80* gene (NM_032504.1:c.4017del:p.(Phe1339Leufs*3)). Her seizures are currently treated with levetiracetam, topiramate, and divalproex sodium.

Case two

This is a two-year-old child, the younger brother of the first case. Had failure to thrive, infantile hypotonia, and psychomotor retardation. She was diagnosed at an earlier stage. Genetic testing revealed a possible pathogenic homozygous frameshift variation in the same gene (NM_032504.1.4017del.(Phe1339Leufs*3)). The boy is presently getting multidisciplinary care to address his developmental problems in addition to comprehensive medical management that includes supportive therapies, including physical and speech therapy. In comparison to his siblings, early identification and intervention are thought to be essential for enhancing his outcomes. It has also been suggested that the family receive more extensive genetic counseling in order to provide them with further support and direction.

Case three

The 17-year-old eldest sibling had a complex medical history, including failure to thrive and a diagnosis of spastic quadriplegic cerebral palsy at birth. The family also reported that he had been admitted for a chest infection three days after birth, leading to multiple hospitalizations for respiratory complications. His epilepsy started at the age of 10 years. In December 2017, when, at the age of 13, he was brought to the emergency room due to generalized tonic-clonic seizures. These seizures lasted for approximately 12 hours, with each episode lasting only a few seconds. Symptoms included excessive drooling, upward rolling of the eyes, and decreased consciousness. His vital signs at the time were concerning: a temperature of 37°C, heart rate of 169 beats per minute, respiratory rate of 29 breaths per minute, and oxygen saturation of 95%. He was treated as per status protocol, which led to stabilization of his seizures. In 2021, the patient developed severe aspiration pneumonia, and his condition worsened, culminating in septic shock and death. Genetic testing had previously confirmed a diagnosis of *UNC80*.

## Discussion

This study expands the understanding of *UNC80* mutations by presenting two novel cases from a Yemeni family, demonstrating severe neurodevelopmental features, including developmental delays, hypotonia, and seizures, consistent with prior reports [4,5]. A review of demographic and genetic data across published cases shows considerable phenotypic variability, with differences in age of onset, severity, and treatment response (Table 1). Differences in genetic background may contribute to phenotypic variability, potentially modifying the clinical expression of *UNC80* mutations.

Most reported patients with *UNC80* mutations are homozygous (24/31) [4,8,9], as observed in these cases. Sanger sequencing commonly identifies these variants in disorders such as infantile hypotonia and severe intellectual disability [6,9], reflecting their impact on the sodium-leak channel complex [6]. Consanguinity, highly prevalent in Middle Eastern populations, raises the likelihood of homozygosity for recessive alleles and is particularly relevant for *UNC80*-related disorders. This pattern was evident in the current family, consistent with elevated consanguinity rates reported in Arab populations [10,11]. The clinical presentation, including cerebral palsy-like symptoms, underscores the importance of genetic screening in consanguineous communities. Both siblings exhibited classic features of *UNC80* deficiency, with homozygous frameshift mutations aligning with previously reported pathogenic mechanisms [7].

The gender distribution in prior cases is nearly equal, with a slight male predominance, though *UNC80* mutations affect both sexes similarly because of their autosomal inheritance. Comprehensive management remains supportive, incorporating anticonvulsants, nutritional and developmental interventions, and targeted therapies for associated complications, consistent with current treatment guidelines [5]. The observed use of prednisolone for seizure control in one case represents an isolated, anecdotal finding, and its relevance to *UNC80*-related disorders remains unclear. This observation should be interpreted with caution, as there is no clear evidence supporting corticosteroid efficacy in this condition, and no therapeutic implications can be inferred at this stage. Furthermore, genetic counseling is essential in managing inherited neurodevelopmental conditions, particularly in consanguineous populations. Early molecular testing and intervention, as demonstrated in the younger sibling, may improve outcomes [12-16].

**Table 1:** Demographic data and genetic background of the reported cases F - female; M - male; NM - not mentioned; y - years; m - months; + indicates the presence of the variable; — indicates the absence of the variable

Variables		Demographic data						Genetic background					
Patient No.	Authors	Gender	Nationality	Ethnicity	Current age	Age group		Allele	Homozygosity	Sanger Sequencing	Consanguinity	Age of diagnosis (seizure onset)	Age group
1	Kelesoglu et al. (2023) [14]	M	NM	NM	6y	Preschool		c.6495G > A:p.Trp2165Ter	Homozygous	NN	+	3y	Toddler
2	Tao et al. (2021) [15]	M	China	Chinese	8y	School age		c.5609-4G>A in the UNC80 gene,	Homozygous	Performed	—	13m	Toddler
3	Stenehjem et al. (2019) [16]	M	NM	NM	3y	Toddler age		UNC80 mutation of 44 Mb homozygosity of 2q33.1-q37.3 and smaller regions of homozygosity on chromosomes two, three, 11, and 16	Homozygous	NM	NM	NM	NM
4	Kuptanon et al. (2019) [17]	F	NM	NM	15 m	Neonate		c.3226C>T (p.Arg1076Ter) and c.3205C>T (p.Arg1069Ter), in UNC80	Heterozygous	NM	—	9m	Neonate
5	Bramswig et al. (2018) [2]	M	Syria	Arab	1y	Infant		UNC80 (NM_032504.1):chr2:g.209972258C > T:GRCh38/hg38:c.8116C > T:p.(Arg2706Ter)	Homozygous	Performed	+	Birth	Neonate
6	Bramswig et al. (2018) [2]	M	Saudi	Arab	3y	Toddler age		UNC80 (NM_032504.1):chr2:g.209777479C > T:GRCh38/hg38:c.520C > T:p.(Arg174Ter)	Homozygous	Performed	+	Birth (4 months)	Neonate
7	Bramswig et al. (2018) [2]	F	Saudi	Arab	1y	Infant		chr2:g.209777479C>T [GRCh38/hg38], c.520C>T p.(Arg174Ter)	Homozygous	Performed	+	Birth	Neonate
8	Bramswig et al. (2018) [2]	F	Saudi	Arab	NM	NM		chr2:g.209777479C>T [GRCh38/hg38], c.520C>T p.(Arg174Ter)	Homozygous	Performed	+	5m	Infant
9	Bramswig et al. (2018) [2]	F	Saudi	Arab	3y	Toddler age		chr2:g.209777479C>T [GRCh38/hg38], c.520C>T p.(Arg174Ter)	Homozygous	Performed	+	Birth	Neonate
10	Bramswig et al. (2018) [2]	M	France	NM	7y	School age		chr2:g.209825974delT [GRCh38/hg38], c.2399delT,	Homozygous	Performed	—	Early life	NM
11	Bramswig et al. (2018) [2]	M	Egypt	Arab	10y	School age		chr2:209817940_209817941delAC [GRCh38/hg38], c.1681_1682delAC, p.(Thr561Argfs*33)	Homozygous	Performed	+	Early life	NM
12	Bramswig et al. (2018) [2]	M	Egypt	Arab	3.8y	Toddler age		chr2:209817940_209817941delAC [GRCh38/hg38], c.1681_1682delAC, p.(Thr561Argfs*33)	Homozygous	Performed	+	8m	Infant
13	Bramswig et al. (2018) [2]	F	Egypt	Arab	3y	Toddler age		chr2:g.209929933C>T [GRCh38/hg38], c.5671C>T, p.(Arg1891Ter)	Homozygous	Performed	+	2m	Infant
14	Bramswig et al. (2018) [2]	M	Egypt	Arab	1.9y	Toddler age		chr2:g.209970959T>G [GRCh38/hg38], c.8058+2T>G, p.?	Homozygous	Performed	+	NM	NM
15	Bramswig et al. (2018) [2]	F	Egypt	Arab	10y	School age		chr2:g.209786065G>A [GRCh38/hg38], c.601-1G>A, p.?	Homozygous	Performed	+	NM	NM
16	Bramswig et al. (2018) [2]	M	Egypt	Arab	9.5y	School age		chr2:g.209786065G>A [GRCh38/hg38], c.601-1G>A, p.?	Homozygous	Performed	+	NM	NM
17	Hong et al. (2018) [5]	M	NM	NM	8y	School age		NM	NM	NM	NM	NM	NM
18	He et al. (2018) [12]	M	China	Chinese	9.5y	School age		c.3719G > A (p.W1240*)/c.4926_4937del(p.N1643_L1646del)	Heterozygous	Performed	—	2y	Toddler
19	He et al. (2018) [12]	F	China	Chinese	5.8y	Preschool		c.3719G> A (p.W1240*)/c.4926_4937del (p.N1643_L1646del)	Heterozygous	Performed	—	NM	NM
20	Obeid et al. (2018) [3]	F	Palestinian-Emirati	Arab	13y	Adolescent		UNC80:c.8525G>A:Exon 56(p.Arg2842Gln)	Homozygous	Performed	+	8m	Infant
21	Shamseldin et al. (2016) [4]	M	Saudi	Arab	6.5y	School age		UNC80 (NM_032504.1):c.3793C>T:(p.Arg1265)	Homozygous	Performed	+	4.5y	Toddler
22	Shamseldin et al. (2016) [4]	M	Saudi	Arab	2y	Infant		UNC80 (NM_032504.1):c.3793C>T:(p.Arg1265)	Homozygous	Performed	+	NM	NM
23	Shamseldin et al. (2016) [4]	M	Saudi	Arab	13m	Toddler age		UNC80 (NM_032504.1):c.3793C>T:(p.Arg1265)	Homozygous	Performed	+	13m	Toddler
24	Shamseldin et al. (2016) [4]	F	Saudi	Arab	7y	School age		UNC80 (NM_032504.1):c.1078C>T:(p.Arg360)	Homozygous	Performed	+	5.5m	Infant
25	Shamseldin et al. (2016) [4]	F	Egyptian	Arab	8y	School age		UNC80 (XM_005246476.1):c.565G>A:(p.Val189Met)	Homozygous	Performed	+	8y	School age
26	Shamseldin et al. (2016) [4]	F	Egyptian	Arab	4y	Preschool		UNC80 (XM_005246476.1):c.565G>A:(p.Val189Met)	Homozygous	Performed	+	4y	Preschool
27	Stray-Pedersen et al. (2016) [9]	F	Iraq	Arab	4y	Preschool		UNC80 (NM_032504.1):chr2:210783340C>T:c.5098C>T:Exon 32:p.Pro1700Ser	Homozygous	Performed	+	3m	Infant
28	Stray-Pedersen et al. (2016) [9]	F	Morocco	Arab	4y	Preschool		UNC80 (NM_032504.1):chr2:210824431G>C:c.7607G>C:Exon 50:p.Arg2536Thr	Homozygous	Performed	+	3y and 10m	Toddler
29	Stray-Pedersen et al. (2016) [9]	F	Norway	Norwegians	15y	Adolescent		UNC80 (NM_032504.1):chr2:210832310T>A:c.7757T>A:Exon 51:p. Leu2586	Heterozygous	Performed	—	6m	Infant
30	Stray-Pedersen et al. (2016) [9]	F	Norway	Norwegians	10y	School age		UNC80 (NM_032504.1):chr2:210685105delA:c.2033delA:Exon 13:p.Asn678Thrfs*15	Heterozygous	Performed	—	3y	Toddler
31	Hong et al. (2018) [8]	M	NM	NM	8y	School age		NM	NM	NM	NM	NM	NM

The structural findings from Table 2 indicate that 40% of the patients had documented brain abnormalities, with 32.3% having microcephaly. A number of studies have shown the link between neurological developmental issues and growth abnormalities like microcephaly [6]. showed that significant global developmental delays are often associated with microcephaly in children, particularly among those with genetic mutations like *UNC80*. This association is further supported by a study that reported that patients with microcephaly are more prone to experiencing intellectual disability, hypotonia, and feeding difficulties [4]. Extract from Table 2 shows the absence of macrocephaly, and a higher incidence of microcephaly in this table provides credibility to the concept that postnatal brain growth failure is a significant factor in the etiology of *UNC80*-related diseases [7]. It is also noted that microcephaly is a critical trait in *UNC80* mutations and can occur without any obvious brain deformities. This emphasizes the necessity of genetic testing and extensive clinical examinations in children with neurodevelopmental delays, even when neuroimaging does not indicate any substantial abnormalities. In Table 2, behavioral problems were noted in 60% of the patients.

**Table 2:** Anthropometric measurement, seizure semiology, and neuroradiological characteristics of the reported cases GTC - generalized tonic-clonic seizure; NM - not mentioned; FTT - failure to thrive; PT: NA - not applicable preterm; + indicates the presence of the variable; — indicates the absence of the variable

Variables		Birth parameters				Current parameters			Brain abnormalities			Clinical characteristics						
Patient No.	Authors	Birth time	Weight (kg)	Height (cm)	Head circumference	Weight (kg)	Height (cm)	Head circumference (cm)	Semiology	Microcephaly	Macrocephaly	Infantile hypotonia	Muscular disorder	Behavioral problems	Head and face deformities	Eyes abnormalities	Ear abnormalities	
1	Kelesoglu et al. (2023) [14]	FTT	19	111	49	19	111	49	Focal febrile, GTC, recurrent status epilepticus	—	—	—	—	—	—	—	—	
2	Tao et al. (2021) [15]	FTT	3.15	51	36	15.5	107	47.1	No seizure	+	—	+	+	+	+	+	+	
3	Stenehjem et al. (2019) [16]	NM	NM	NM	NM	NM	NM	NM	NM	NM	NM	+	NM	NM	NM	NM	NM	
4	Kuptanon et al. (2019) [17]	PT	2.2	NM	NM	5.5	69	41.5	Myoclonic	NM	NM	+	+	NM	+	NM	NM	
5	Bramswig et al. (2018) [2]	FTT	3.5	NM	NM	6.5	NM	44.5	No seizure	NM	NM	+	+	NM	+	+	+	
6	Bramswig et al. (2018) [2]	FTT	2.23	45	33	3.3	58	39	Tonic-clonic	NM	NM	+	+	+	+	+	+	
7	Bramswig et al. (2018) [2]	FTT	2.24	45	NM	3.9	66	41.6	No seizure	+	—	+	+	+	+	+	+	
8	Bramswig et al. (2018) [2]	FTT	2.1	44	30.5	NM	NM	NM	No seizure	+	—	+	—	—	+	—	+	
9	Bramswig et al. (2018) [2]	PT	2.4	46	31	NM	NM	NM	No seizure	+	—	+	+	—	+	+	+	
10	Bramswig et al. (2018) [2]	FTT	3.08	48	35	13.4	99	51	No seizure	NM	NM	+	+	—	+	+	+	
11	Bramswig et al. (2018) [2]	FTT	3.2	50	34.5	16	114	49.5	No seizure	NM	NM	—	+	+	+	+	+	
12	Bramswig et al. (2018) [2]	FTT	3.4	49	34	12	92	47	No seizure	NM	NM	—	+	+	+	+	+	
13	Bramswig et al. (2018) [2]	FTT	3	48	33.3	4.6	68	37	No seizure	NM	NM	+	+	+	+	+	+	
14	Bramswig et al. (2018) [2]	FTT	3.5	50	34.3	9.1	87	46	No seizure	NM	NM	+	+	+	+	+	+	
15	Bramswig et al. (2018) [2]	FTT	2.8	47	33	14	105.5	48	No seizure	NM	NM	+	+	—	+	+	+	
16	Bramswig et al. (2018) [2]	FTT	3	50	34.2	16	110	51	No seizure	NM	NM	+	+	—	+	+	+	
17	Hong et al. (2018) [5]	NM	NM	NM	NM	NM	NM	NM	Refractory epilepsy	NM	NM	NM	NM	+	NM	NM	NM	
18	He et al. (2018) [12]	NM	3.35	50	NM	25	135	51	Unspecified seizure	—	—	+	—	+	+	+	—	
19	He et al. (2018) [12]	NM	3.1	NM	NM	NM	100	NM	—	+	—	+	—	+	+	+	—	
20	Obeid et al. (2018) [3]	38	2.6	NM	33	40.2	144	51.5	Febrile convulsion	+	NM	+	NM	+	+	+	+	
21	Shamseldin et al. (2016) [4]	NM	3	46	NA	NM	NM	NM	No seizure	+	NM	+	NM	+	+	+	NM	
22	Shamseldin et al. (2016) [4]	NM	2.7	51	36	4.8	79	43	No seizure	+	NM	+	NM	NM	+	+	NM	
23	Shamseldin et al. (2016) [4]	NM	2.9	53	34	5.13	71	43	No seizure	—	NM	+	NM	NM	+	NM	NM	
24	Shamseldin et al. (2016) [4]	NM	2.6	53	34	13.45	109.5	48.5	No seizure	+	NM	+	NM	NM	+	+	NM	
25	Shamseldin et al. (2016) [4]	NM	2.8	50	34.5	18.5	112	50	Unspecified seizure	—	NM	+	NM	+	+	NM	+	
26	Shamseldin et al. (2016) [4]	NM	3	51	34	14	NM	47.5	No seizure	—	NM	+	NM	+	+	NM	+	
27	Stray-Pedersen et al. (2016) [9]	41	3	NM	33	7.4	72	43	GTC	NM	NM	+	NM	NM	+	+	NM	
28	Stray-Pedersen et al. (2016) [9]	41	3.158	NM	NA	12.9	91.5	47	GTC	NM	NM	+	NM	NM	+	+	NM	
29	Stray-Pedersen et al. (2016) [9]	38	2.960	NM	32	40.2	144	51.5	Myoclonic, atonic, GTC, atypical absences	NM	NM	+	NM	NM	+	+	NM	
30	Stray-Pedersen et al. (2016) [9]	40	3.070	NM	35	23.5	125	50.5	Atonic, atypical absences	NM	NM	+	NM	NM	+	+	NM	
31	Hong et al. (2018) [8]	NM	NM	NM	NM	NM	NM	NM	Unspecified seizure	NM	NM	NM	NM	NM	NM	NM	NM	

These disturbances typically took the form of irritability and difficulties controlling their emotions. Behavioral challenges are often found in children with the *UNC80* gene, as reported by Bramswig et al. [7], who reported similar findings in patients with severe neurodevelopmental delay. Moreover, almost all cases reviewed in the literature exhibited global developmental delays, with 28 out of 31 [8,9] individuals demonstrating such delays, alongside motor delays encompassing both gross and fine motor skills (27/31 and 25/31, respectively) [4,8,9]. This observation aligns closely with our three reported cases, which also exhibited similar developmental and motor delays. Furthermore, a significant proportion of individuals in the literature presented with a history of failure to thrive (20/31) [3,4,8,9,12] and feeding difficulties (17/31) [2-5,8,9,12]. Conversely, fewer published cases reported cognitive (12/31) [2-5,8,9,12] and social delays (11/31) [2-5,8,9,12]. In our three cases, we noted generalized developmental delays accompanied by positive symptoms consistent with previous reports, as summarized in Table 3.

**Table 3:** Developmental and electrophysiological characteristics of the reported cases NM - not mentioned; + indicates the presence of the variable; — indicates the absence of the variable.

Patient No.	Authors	Developmental delay (28/31)	Cognitive (12/31)	Gross motor delay (27/31)	Fine motor delay (25/31)	Speech delay (27/31)	Social delay (11/31)	Feeding difficulity (17/31)	Failure to thrive (20/31)	EEG abnormities (N=11 /Ab=13 / NM=7)	MRI abnormities (11/31)
1	Kelesoglu et al. (2023) [14]	—	—	—	—	—	—	NM	—	NM	NM
2	Tao et al. (2021) [15]	+	NM	+	+	+	+	NM	+	Normal	Bilateral dilation of lateral ventricles,
3	Stenehjem et al. (2019) [16]	+	NM	NM	NM	NM	NM	+	+	Abnormal	NM
4	Kuptanon et al. (2019) [17]	+	NM	+	NM	NM	NM	+	+	Abnormal	Normal
5	Bramswig et al. (2018) [2]	+	NM	+	+	+	NM	—	+	Normal	Normal
6	Bramswig et al. (2018) [2]	+	NM	+	+	+	NM	+	+	Abnormal	Diffusion restriction in both parietooccipital cortex
7	Bramswig et al. (2018) [2]	+	NM	+	+	+	NM	+	+	Normal	NM
8	Bramswig et al. (2018) [2]	+	NM	+	+	+	NM	+	+	NM	Small cystic formation at inferior aspect of the right basal ganglia representing likely prominent perivascular space. Likely no clinical significance.
9	Bramswig et al. (2018) [2]	+	NM	+	+	+	NM	+	+	Normal	NM
10	Bramswig et al. (2018) [2]	+	NM	+	+	+	NM	+	+	Normal	Normal
11	Bramswig et al. (2018) [2]	+	NM	+	+	+	NM	+	+	Normal	Mild cortical changes, thin corpus callosum.
12	Bramswig et al. (2018) [2]	+	NM	+	+	+	NM	+	+	Normal	Mild cortical changes, thin corpus callosum.
13	Bramswig et al. (2018) [2]	+	NM	+	+	+	NM	+	+	Normal	Thin corpus callosum.
14	Bramswig et al. (2018) [2]	+	NM	+	+	+	NM	+	+	Abnormal	Mild cortical changes, thin corpus callosum.
15	Bramswig et al. (2018) [2]	+	NM	+	+	+	NM	+	+	Normal	Mild cortical changes, thin corpus callosum.
16	Bramswig et al. (2018) [2]	+	NM	+	+	+	NM	+	+	normal	Mild cortical changes, thin corpus callosum.
17	Hong et al. (2018) [5]	+	NM	NM	NM	+	NM	+	NM	NM	NM
18	He et al. (2018) [12]	+	+	+	+	+	NM	—	—	abnormal	NM
19	He et al. (2018) [12]	NM	+	+	+	+	NM	—	+	normal	Normal
20	Obeid et al. (2018) [3]	+	—	+	—	+	—	—	—	Abnormal	Normal
21	Shamseldin et al. (2016) [4]	+	+	+	+	+	+	NM	NM	NM	mild diffuse brain atrophy
22	Shamseldin et al. (2016) [4]	+	+	+	+	+	+	NM	NM	Abnormal	Normal
23	Shamseldin et al. (2016) [4]	+	+	+	+	+	+	NM	+	NM	Normal
24	Shamseldin et al. (2016) [4]	+	+	+	+	+	+	NM	+	Abnormal	Normal
25	Shamseldin et al. (2016) [4]	+	+	+	+	+	+	NM	+	Abnormal	NM
26	Shamseldin et al. (2016) [4]	+	+	+	+	+	+	NM	+	NM	NM
27	Stray-Pedersen et al. (2016) [9]	+	+	+	+	+	+	+	NM	Abnormal	thin corpus callosum
28	Stray-Pedersen et al. (2016) [9]	+	+	+	+	+	+	+	NM	Abnormal	Normal
29	Stray-Pedersen et al. (2016) [9]	+	+	+	+	+	+	—	NM	Abnormal	Normal
30	Stray-Pedersen et al. (2016) [9]	+	+	+	+	+	+	+	NM	Abnormal	Normal
31	Hong et al. (2018) [8]	NM	NM	NM	NM	NM	NM	NM	NM	NM	NM

These findings suggest a pattern in the clinical presentation of *UNC80* mutations, reinforcing the need for comprehensive assessment in affected individuals. *UNC80* mutations are associated with a distinct clinical phenotype characterized by significant developmental and motor delays, as well as feeding difficulties, reflecting underlying neurodevelopmental disruptions. The high incidence of global developmental delays in our cases, consistent with the literature, global developmental delay, a symptom as manifested from static encephalopathy, has been associated with affected individuals [4]. Additionally, the consistent observation of motor delays may be linked to loss-of-function mutations of NALCN due to *UNC80* being a large component of the NALCN sodium-leak channel complex that regulates the basal excitability of the nervous system, and loss-of-function mutations of NALCN cause a psychomotor delay with infantile hypotonia [13-17]. Moreover, the prevalence of feeding difficulties among affected individuals due to oral motor dysfunction causes problems in chewing, swallowing, and oral coordination, leading to feeding difficulties [7].

One limitation in this research is the absence of extended follow-up data for the patients. The availability of thorough clinical records is restricted by the temporary presence of our patient population, often caused by migratory influences. Therefore, we lack specific details regarding patients' seizure characteristics, electroencephalogram (EEG) results, and MRI findings. Additionally, because of these limitations, we cannot collect comprehensive clinical information on siblings affected, which could have offered valuable information on genetic influences or familial patterns impacting seizure characteristics.

## Conclusions

In conclusion, the identification of three novel homozygous *UNC80* mutations in this Yemeni family broadens our understanding of *UNC80*-related neurodevelopmental disorders and underscores the profound clinical impact of this gene on early development. These cases highlight the critical role of early genetic diagnosis in guiding tailored interventions, informing multidisciplinary care, and optimizing long-term outcomes.

They also emphasize the importance of genetic screening in populations with high rates of consanguinity, where autosomal recessive disorders are more prevalent. Moreover, documenting these cases contributes to the growing global understanding of *UNC80* deficiency and its variable presentations. Future studies, including functional analyses and investigations in larger cohorts, are essential to further elucidate disease mechanisms and explore potential targeted therapies.
